# Picornavirus RNA is protected from cleavage by ribonuclease during virion uncoating and transfer across cellular and model membranes

**DOI:** 10.1371/journal.ppat.1006197

**Published:** 2017-02-06

**Authors:** Elisabetta Groppelli, Hazel C. Levy, Eileen Sun, Mike Strauss, Clare Nicol, Sarah Gold, Xiaowei Zhuang, Tobias J. Tuthill, James M. Hogle, David J. Rowlands

**Affiliations:** 1 School of Molecular and Cellular Biology & Astbury Centre for Structural Molecular Biology, Faculty of Biological Sciences, University of Leeds, Leeds, West Yorkshire, United Kingdom; 2 Department of Biological Chemistry and Molecular Pharmacology, Harvard Medical School, Boston, Massachusetts, United States of America; 3 Program in Virology and Department of Chemistry and Chemical Biology, Harvard University, Cambridge, Massachusetts, United States of America; 4 The Pirbright Institute, Pirbright, Surrey, United Kingdom; 5 Howard Hughes Institute and Department of Chemistry and Chemical Biology, Harvard University, Cambridge, Massachusetts, United States of America; Stanford University, UNITED STATES

## Abstract

Picornaviruses are non-enveloped RNA viruses that enter cells via receptor-mediated endocytosis. Because they lack an envelope, picornaviruses face the challenge of delivering their RNA genomes across the membrane of the endocytic vesicle into the cytoplasm to initiate infection. Currently, the mechanism of genome release and translocation across membranes remains poorly understood. Within the enterovirus genus, poliovirus, rhinovirus 2, and rhinovirus 16 have been proposed to release their genomes across intact endosomal membranes through virally induced pores, whereas one study has proposed that rhinovirus 14 releases its RNA following disruption of endosomal membranes. For the more distantly related aphthovirus genus (e.g. foot-and-mouth disease viruses and equine rhinitis A virus) acidification of endosomes results in the disassembly of the virion into pentamers and in the release of the viral RNA into the lumen of the endosome, but no details have been elucidated as how the RNA crosses the vesicle membrane. However, more recent studies suggest aphthovirus RNA is released from intact particles and the dissociation to pentamers may be a late event. In this study we have investigated the RNase A sensitivity of genome translocation of poliovirus using a receptor-decorated-liposome model and the sensitivity of infection of poliovirus and equine-rhinitis A virus to co-internalized RNase A. We show that poliovirus genome translocation is insensitive to RNase A and results in little or no release into the medium in the liposome model. We also show that infectivity is not reduced by co-internalized RNase A for poliovirus and equine rhinitis A virus. Additionally, we show that *all* poliovirus genomes that are internalized into cells, not just those resulting in infection, are protected from RNase A. These results support a finely coordinated, directional model of viral RNA delivery that involves viral proteins and cellular membranes.

## Author summary

Picornaviruses are a large family of important human and animal pathogens that include poliovirus, human rhinovirus and foot-and-mouth disease virus. Picornaviruses enter the host cell by hijacking one of the vesicle-mediated cellular entry routes. However, once the virus is internalized, the mechanism used to deliver the viral genome across the vesicle membrane and into the cytoplasm remains unclear and even controversial. Here we show that for poliovirus (a member of the enterovirus genus), viral RNA is translocated directly from the particle, across the vesicle membrane into the lumen of liposomes in a receptor-decorated liposome model, or cytoplasm during infection, without being exposed to external medium surrounding the liposomes or the lumen of the entry vesicle, respectively. Our results suggest that the interaction between the viral particle and the membrane results in a specific mechanism of viral genome delivery that not only directs but also protects the RNA so that it reaches the cytoplasm as an intact and functional molecule. Additionally, we show that this is also the case for equine rhinitis A virus, a member of the aphthovirus genus, whose genome delivery mechanism has previously been thought to differ significantly from the mechanism used by enteroviruses suggesting the possibility of a unified mechanism of RNA delivery for the entire picornavirus family.

## Introduction

Poliovirus (PV) is the type member of the enterovirus genus of the picornavirus family of single stranded RNA viruses and was until recently a major cause of human paralytic disease. Although global vaccination campaigns have largely controlled the incidence of poliomyelitis, PV continues to provide a valuable model system for understanding the molecular biology and pathogenesis of newly emerging pathogenic enteroviruses, such as EV71 [1]. The PV particle is icosahedral, 30 nM in diameter, formed from 60 copies of each of 4 capsid proteins; VP1, 2 and 3 form the icosohedral shell of the particle while VP4 (which is N-terminally myristoylated) [2] and N-terminal extensions of VP1, VP2, and VP3 are disposed as a network on the inner surface [3]. The outer surface of the virus is dominated by star-shaped mesas at the fivefold axes, and three-bladed propeller-like features surrounding the threefold axes. These prominent features are separated by deep grooves surrounding the fivefold mesas, often referred to as the canyon [3,4].

Poliovirus infection begins with the attachment of the virus its receptor CD155/PVR [5]. CD155 is a is a type one glycoprotein comprising three immunoglobulin-like domains, a transmembrane domain and a cytoplasmic domain and normally functions as an adhesion receptor [6,7]. The distal (N-terminal) Ig-like domain of the PVR binds within the PV canyon [8,9]. At physiological temperature the receptor catalyzes [10] a conversion of the virus (160S particle) to an altered particle called the 135S or A particle [11]. This conversion is associated with an expansion of the capsid by about 4%, [12] loss of a fatty acid-like ligand at the base of the canyon [13] and the externalization two normally internal peptides including the myristoylated protein VP4 [11] and the N-terminal extension of VP1 [14], both of which then insert into the membrane [14–16]. The 135S particle is then released from the receptor but remains tethered to the cell membrane by the membrane embedded N-terminal extension of VP1. After conversion, the 135S particle is internalized by a noncanonical endocytic pathway that is independent of vesicle acidification, clathrin, caveolin, flotillin and microtubules, but requires actin and an as yet uncharacterized tyrosine kinase[17]. Although the vesicles are distinct from the more commonly known early and late endosome, we use the general term endosome to describe the compartment. Shortly after the internalization of the 135S particle, an unknown trigger initiates the release of the viral RNA genome, which traverses the endocytic membrane to enter the cytoplasm.

Structures have been determined for a number of the key intermediates [8,13,18–23] these structures along with genetic and biochemical studies [12,15,22,24] provide evidence that suggests that the released VP4 protein, together with the externalised N-terminal sequences of VP1, form a membrane-spanning channel through which the RNA is transported into the cytoplasm. It seems reasonable to hypothesize that the close proximity (interaction) of the viral proteins and the bilayer could provide a localized and protected environment that shields the viral RNA from the content of the endocytic vesicle. This would be of critical importance for maintenance of virus infectivity, because RNases are present in serum and are known to be internalized within endocytic vesicles [25]. Enterovirus particles are icosohedral and there is at present no clear mechanism for polarization of RNA release at a point on the particle adjacent to the endosomal membrane. A possible explanation is that engagement with the receptor induces a conformational polarity on the virus particle such that the RNA is released at that point [22]. However, given the high particle to infectious unit ratios typical of picornaviruses, it is formally possible that a high proportion of virions simply sacrifice the genomes that are released from the particles at sites distal from the point of association with the membrane and are therefore ‘lost’ into the vesicle lumen. We are thus left with a number of questions: a) is the RNA released from random positions on the particle, only a random <10% of which are adjacent to the membrane? b) does the attachment process induce a polarisation of the particle so that RNA is only released from a position adjacent to the membrane? c) can RNA released into the endosomal lumen traverse the membrane to reach the cytoplasm? d) is the RNA protected during transmission across the membrane from RNases that might be present in the endosomal lumen?

PV is typical of all enteroviruses in that the virus particle remains intact, albeit modified, during the cell infection process and may function as a protective RNA delivery capsule. However, for viruses in the aphthovirus genus (e.g. foot-and-mouth disease virus, FMDV; equine rhinitis A virus, ERAV) the cell entry mechanism(s) are even less well understood. Both FMDV and ERAV are internalized by clathrin-mediated endocytosis and endosomal acidification is necessary for virus uncoating and infection [26–30]. *In vitro* studies have shown that at pH values typically encountered in early endosomes the aphthovirus capsid dissociates into pentameric subunits, releasing the RNA and the internal protein VP4 [31,32]. Although this supports an aphthovirus uncoating model in which acidic pH triggers RNA release, it provides no insight into the mechanism of RNA translocation across the endosomal membrane or how RNA released into the endosomal lumen might be protected from damage in this potentially hostile environment. It is difficult to envision a mechanism that allows the viral RNA to transit the endosomal membrane in an organised fashion without a coordinated involvement of the viral proteins. However, the transient formation of altered particles of ERAV *in vitro* has been described [31]. Interestingly, these altered particles lack RNA and may represent uns and transient forms functionally equivalent to the enterovirus ‘genome delivery capsules’. In this scenario, ERAV might use the same general mechanism for protected genome delivery as PV, with the difference that aphthovirus empty particles are less s than PV 80S and dissociate into pentamers shortly after RNA release.

Here we investigate PV uncoating and RNA delivery strategies using a combination of cell culture and cell-free assays, which together show that the transfer of PV RNA across endosomal membranes or liposome model membranes is unaffected by high concentrations of RNase A. Furthermore, we present evidence to suggest that the genome of the aphthovirus ERAV is similarly protected from RNase present in the endosome during the infection process, in a mechanism reminiscent of that of the enteroviruses.

## Results

### Receptor-decorated liposomes support RNA translocation

**Fig 1:**
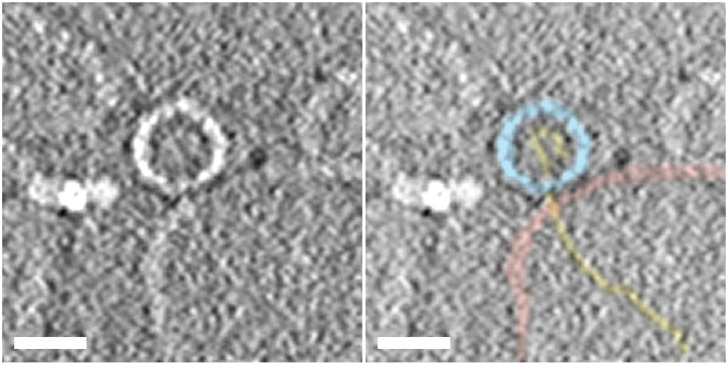
Section through a subtomograms from a cryoelectron tomographic reconstruction of a warmed virus-receptor- liposome complex showing RNA being translocated across the liposome membrane. The samples were produced by heating virus-receptor-liposome complexes at 37°C for 4 min, mixed with colloidal gold, placed on carbon-coated Quantifoil holey grids and flash frozen, and cryo tomographic data were acquired and processed as in [22] The central section through a representative subtomogram containing a single complex from this data set is presented to summarise the path of the viral RNA from the interior of the virus, across the liposome membrane, and into the lumen of the liposomes during uncoating. The left panel shows a section through raw averaged subtomogram showing a virus particle (center) attached to a liposome (bottom right), with density for the RNA clearly extending from the middle of the particle across the membrane and into the lumen of the liposome. The bright feature to the left of the virus is a colloidal gold particle. The right panel shows the same section of the tomogram segmented to highlight the virus capsid (light-blue), the membrane bilayer (pink), and the RNA (gold). The scale bar in both panels is 25 nm.

Previous studies have introduced a simple model system in which liposomes containing low levels of lipids with NiNTA head groups are decorated with the ectodomain of the PVR containing a C-terminal (membrane proximal) six-histidine tag. These receptor-decorated liposomes (RDL) were shown to bind virus and at physiological temperatures induce conformational rearrangements in the virion, externalization of VP4 and the N-terminal region of VP1, the insertion of these externalized peptides into the membrane, and viral RNA release from the protein shell, closely mimicking the steps that occur in the early stages of infection [16]. A structure obtained by averaging subtomograms of individual particles from a large tomographic reconstruction of the complex formed upon warming virus-receptor-liposome complexes to physiological temperature was recently reported, revealing the presence of one or more long umbilical connections linking the virus particle to the surface of the membrane [22]. The raw subtomograms from the tomographic reconstruction of the complexes showed that the altered virus particles contained variable levels of RNA, and in a significant number of the subtomograms containing individual complexes there was clear evidence for RNA being translocated across intact membranes [22]. It should be noted that although not all of the subtomograms in the data set clearly showed RNA entering the liposome, the frequency is significant given that the ability to see RNA depends the timing of RNA release (some particles may not yet have started, some may be complete) and requirement that the plane of the central section contains both center of the virus and the center of the liposome in order for RNA to be seen. More recently cryoelectron micrographs of similar complexes of PV with large (50 nm) receptor-decorated nanodiscs have also shown RNA crossing the membrane in favourable views, and some of these micrographs also show structures in the disc membranes that may be pores [33]. Fig 1 shows a central section through a representative subtomogram from the large tomographic data set of the warmed virus-receptor-liposome complex [22], where the RNA can be clearly followed from the inside to the across the liposome membrane and entering the lumen of the liposome. The RNA that is visible crossing the membrane and in the lumen of the liposome in this subtomogram clearly appears to be linear and therefore almost certainly single-stranded, because RNA secondary structure would be expected to be highly branched in appearance. As noted in the original description of the tomographic structure [22], this would require that the RNA at least transiently loses secondary structure upon release. The loss of secondary structures during uncoating has been reported previously in the live cell imaging studies of PV entry, which showed that the Syto82 dye (an intercalating dye) used to visualize RNA inside virus particles was stripped from the virus particle during RNA release [17]. Loss of RNA secondary structure during uncoating is also consistent with previous studies that showed that PV with neutral red attached to the genome becomes insensitive to light upon RNA release [15,17,24,34,35]. Note, that in other subtomograms in the tomographic reconstruction of this complex [22], in conventional cryoEM images of the corresponding complexes in the receptor-decorated nanodiscs model [33], and in conventional and tomographic cryoreconstructions of heated particles in the absence of membrane that were caught in the act of RNA release [20], the RNA density suggests that the RNA is linear as released but then refolds.

### PV RNA translocation into receptor-decorated liposomes is insensitive to RNase A

**Fig 2:**
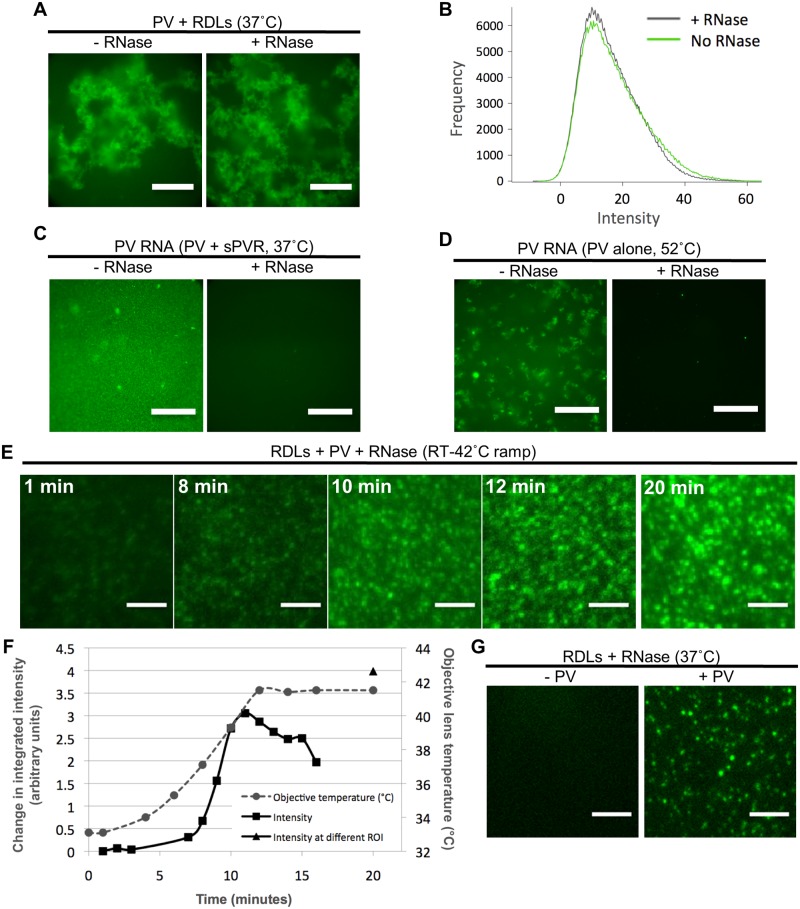
Receptor-decorated liposomes containing fluorescent dye detect PV RNA release. **A)** Representative images of YoPro-1 encapsulating receptor-decorated liposomes (YRDLs) complexed with PV in the presence or absence of RNase A (50 μg/ml). Note that RNase A was added to the extra-liposomal space after PV-YRDL complexes were formed, but prior to heating the samples for 20 min at 37°C. Images were collected at room temperature using a 20X objective as described in Materials and Methods. Scale bars are 200 μm. **B)** Normalized histograms showing the number of pixels (y-axis) with a given level of fluorescence (in arbitrary units) (x-axis) of PV-YRDL complexes shown in A in the absence (green curve) and presence (black curve) of RNase A. (**C and D)** Representative images of PV RNA (in the absence of liposomes) in the presence of YoPro-1 dye following induction of uncoating by sPVR at 37°C (C) or by heating at 52°C (D) for 20 min in the presence or absence of RNase A (50 μg/ml). Images were collected using a 100X objective as described in Materials and Methods. Scale bars are 40 μm. **E)** Representative still frames from a time lapse of PV-YRDLs gradually heated from room temperature to 42°C. Average time lapse for averaged image is indicated. After 15 min of imaging a single field of view, a second region of interest was imaged in order to evaluate the influence of photobleaching on the fluorescence intensity (second ROI at 20 min shown on the right-side panel). Images were captured at 100x magnification using a custom built Total Internal Reflectance Fluorescence Microscopy (TIR-FM) setup, attached to an Olympus IX-71 microscope, as described in Material and Methods. Scale bars are 5 μm. **F)** PV-YRDLs integrated fluorescence intensity obtained as indicated in Materials and Methods (expressed as fold change of T = 1 min, left y-axis) during a 20 min time course (time in min along the x-axis) when the sample was heated from room temperature to 42°C. The temperature of the lens (right y-axis) is shown as a function of time (grey dashed line). Because the objective lens and the sample are 1.18 mm apart (with oil connecting the lens to the sample slide), the temperature of the lens is used to estimate the temperature of the sample. 42°C is the upper limit of the imaging apparatus. The black triangle shows the integrated fluorescence intensity of a region of interest that was imaged at a single time point of 20 min in order to assess photobleaching. **G)** Representative images of YoPro-1 encapsulating RDL using the same microscope setup described for E. YRDLs were incubated at 37°C for 10 min, alone with no PV (left), or were pre-incubated with PV at room temperature for 10 min to allow complex formation, and then incubated 37°C for 10 min (right). Scale bars are 5 μm.

In order to study the mechanism of RNA release from the PV capsid in the context of a membrane tethered receptor, a real-time, fluorescence and liposome-based method was developed that allowed detection of PV-mediated RNA translocation across a lipid bilayer. YoPro-1, which increases fluorescence upon nucleic acid binding, was captured inside the lumen of the liposomes, and was also present outside of liposomes. RNase A was added (except when noted) to a final concentration of 50 μg/ml to the preformed PV-RDL complexes. Thus, the liposome interior space remained RNase A free. Upon heat-treatment of complex at 37°C, similar levels of YoPro-1 fluorescence due to RNA binding were detected in the absence (left panel) or presence (right panel) of RNase A (Fig 2A). The fluorescence in each case was quantified constructing normalized histograms of the frequency of occurrence (y-axis) of a given level of relative fluorescence (x-axis) within a masked area of each image that contained significant signal as described in Materials and Methods (Fig 2B). The striking similarity in the fluorescent in the absence (green line) or presence (black line) of RNase A strongly suggests that virtually all of the RNA that is released is transported into the lumen of the liposome, and little or none of the RNA is released into the surrounding medium. Note that the large number of pixels within the masked areas of the images allows the histogram to be sampled on a very fine grid. The low deviations each data point from a smooth curve that could be fit to the histogram provide estimates of the error in each data point. To test the sensitivity of PV RNA to RNase A in the absence of RDLs, PV RNA was liberated from the protein shell by warming PV in the presence of soluble PVR at 37°C for 20 min (Fig 2C) and by heating free virus at 52°C for 20 min. (Fig 2D), in the absence (Fig 2C and 2D left panels) or presence of RNase A (Fig 2C and 2D right panels) of RNase A. Addition of RNase A to the PV RNA abolished fluorescence, substantiating that PV RNA is fully susceptible to degradation by RNase A. Thus, in the presence of RNase A, RNA is only detectible by liposome-sequestered dye, and not by the dye that remains outside of the liposomes. Upon gradual temperature ramping of the PV-RDL complexes from RT to 42°C (Fig 2E, S1 Movie), the integrated fluorescence intensity, which is correlated to the number of binding events between YoPro-1 molecules and RNA in a defined area, increased over time (Fig 2F).

In the absence of RNA and PV, minimal fluorescence was detected in these samples at room temperature (RT) or when incubated for 10 min at 37°C (Fig 2G, left panel). To visualize individual liposomes, PV was added in the presence of RNase A, and the sample was diluted 1:5 (Fig 2G, right panel). Fluorescence was confined to individual points (liposomes) with no fluorescence between points, demonstrating that translocation into the liposomes protects RNA from RNase degradation. In conclusion, we have successfully developed an uncoating *in vitro* assay that shows that PV particles can translocate their RNA directly across a lipid membrane into the lumen of a model vesicle. We have used this model to show that this transfer is protected from RNase digestion, and have shown that this release is highly directional with little or no release of RNA into the surrounding medium.

### PV infectivity is unaffected by the presence of RNase A in the infecting medium

**Fig 3:**
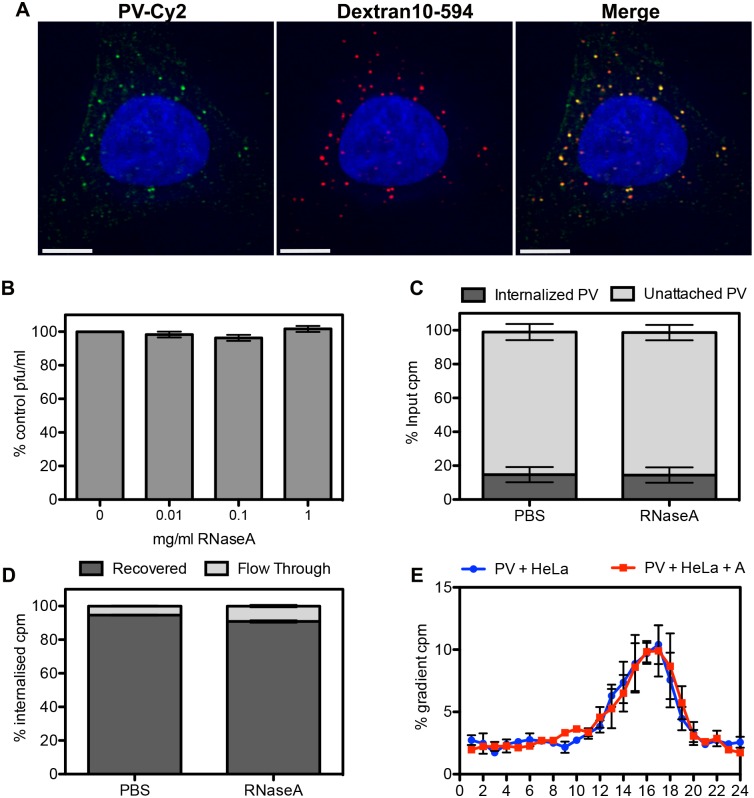
PV infectivity and RNA integrity are not affected by the presence of high levels of RNase A during the infection process. **A)** Representative image of HeLa Ohio cells infected with PV-Cy2 (green) in the presence of Dextrans-10 kDa conjugated to Alexa-594 (red) fixed 15 min post-infection. The degree of co-internalization (right-hand side panel) was measured on 10 random cells, R = 0.89 +/- 0.09 (SD). Scale bar is 5 μm. **B)** Plaque assay of PV in the presence of 0–1 mg/ml RNase A. Plaque forming units were expressed as percentage of no RNase A control. **C)** Scintillation counting of internalized vs unattached ^3^H-U-PV in HeLa Ohio cells in the presence of RNase A (1 mg/ml) or PBS carrier control. **D)** Scintillation counting of recovered and flow-through samples after a column-based RNA purification procedure of ^3^H-U-PV RNA internalized into HeLa Ohio cells in the presence of RNase A (1 mg/ml) or PBS carrier control. **E)** Scintillation counting of sucrose density gradient (15–30% sucrose, 0.1% SDS, 0.1 M Na acetate. Fraction 1 = top, 15% sucrose) of ^3^H-U-PV RNA recovered from HeLa Ohio cells 30 min post-infection in the presence or absence of 1 mg/ml RNase A (PV+HeLa+A, red line, and PV+HeLa, blue line, respectively). Data is expressed as percentage of the total counts per minutes (cpm) loaded onto the gradient. All data are from three independent experiments and error bars show standard error.

Having established with the liposome-based assay that RNA can be translocated across intact membranes and that the translocated RNA is protected from RNase A during this process, we sought to determine whether the viral genome was similarly protected during the process of infection of cultured cells. We started by infecting HeLa Ohio cells with a single dose of PV (20–30 pfu) in the presence of escalating concentrations of RNase A (0 to 1 mg/ml). After 1 hour the growth media was replaced with agarose containing media and plaques were counted 48 h later. We speculated that RNase A in the medium would be co-endocytosed with virus particles and, since a single cleavage of the RNA would abrogate infectivity, exposure of the RNA to RNase A in the endosome during transit from the 135S delivery particle into the cytoplasm would result in a reduced titre. The results in Fig 3B show that concentrations of RNase A up to 1 mg/ml had no effect on virus titre, suggesting that the genomic RNA is protected from degradation during the infection process.

Although RNase A is most active between pH 6–10, it retains function at pH 5.0 and is, therefore, enzymatically active at early and late endosomal pH. RNase A has also been shown to possesses strong thermal stability (T_m_ = 62°C) [36] and has a long half-life of 55–95 h in mammalian cells, which is at least in part due to RNase A resistance to lysosomal proteolytic degradation [37,38]. We wished to demonstrate that RNase A was indeed co-internalized with virus particles in intracellular vesicles. We used fluorescently labelled dextrans as marker of fluid-phase uptake and infected cells with PV tagged with the fluorescent dye Cy2. After 10–15 min at 37°C, to allow internalisation of the virus, non-attached viral particles were removed by washing and the cells fixed for examination by fluorescence microscopy. Co-localization analysis of 10 random cells resulted in R = 0.89 +/- 0.09 (SD) (Fig 3A), strongly indicating that soluble factors in the infection medium are efficiently co-internalized with the virus.

### PV genome integrity during the infection process is unaffected by RNase A in the infection medium

The pfu/particle ratio for PV is typically approximately 1:100 and so it cannot be concluded that results obtained from infectivity measurements equate to the experience of all, or even the majority, of the viral genomes present in the population. Given the symmetric nature of the picornavirus particle, the RNA could potentially emerge from any of multiple equivalent sites on the particle, only one or few of which are adjacent to the cellular endosomal membrane, while most would open up to the lumen of the entry vesicle. However, it has been postulated that the interaction of the virus with its receptor may induce localized conformational alterations in the particles that result in a ‘polarization’ that allows the RNA to emerge at a point immediately adjacent to the membrane [22]. In this case, it would be expected that all of the endocytosed particles, which must have all interacted with the receptor in order to be internalized, would translocate their RNA across the membrane into the cytoplasm, and no RNA would be exposed to the lumen of the entry vesicle. To test this assumption we followed the fate of RNA from virus that had been metabolically labelled with ^3^H-uridine (^3^H-U). In order to synchronize entry, HeLa Ohio cells were cooled to 4°C for 30 min before ^3^H-U-PV was allowed to attach in normal medium (with carrier control, PBS) or in the presence of 1 mg/ml RNase A for 20 min at 4°C. After attachment, entry and uncoating were allowed by incubation at 37°C for 30 min, since by this time it has been shown that all of the internalized PV particles have converted to empty 80S particles [17,39]. Un-internalized virus was removed by washing with cold PBS and quantified by scintillation counting. Fig 3C shows that up to 20% of the applied radioactive counts (^3^H-U PV) were internalized and that the presence of RNase A in the infection medium did not affect PV internalization. Total RNA was then extracted with Trizol and purified with an RNA binding column. Fig 3D shows that >95% of the internalized radioactive counts were recovered and less than 5% of the counts were collected in the column flow-through, which represents RNA molecules smaller than 20 nt. No statistical difference was detected between the control sample (PBS carrier) and the RNase A sample. Next, the recovered counts were analyzed by sucrose gradient centrifugation. Fig 3E shows that the ^3^H-U counts sediment as a single peak that corresponds to full-length viral RNA (fractions 14–18) with no accumulation of signal at the top of the gradients where smaller RNA molecules (i.e. degradation products) would be expected to sediment. No significant difference was detected between the radioactive profile of RNA extracted from cells infected in normal medium (with PBS carrier control) and the RNA from cells infected in the presence of 1 mg/ml RNase A (Fig 3E). These data suggest that viral RNA does not come into contact with co-internalized RNase A during the uncoating process in HeLa Ohio cells, and together with the liposome experiments described above provide strong support a model in which RNA release is highly directional through unique membrane associated sites, rather than occurring randomly through otherwise equivalent sites.

### PV infectivity is unaffected by covalent linkage of RNase A

**Fig 4:**
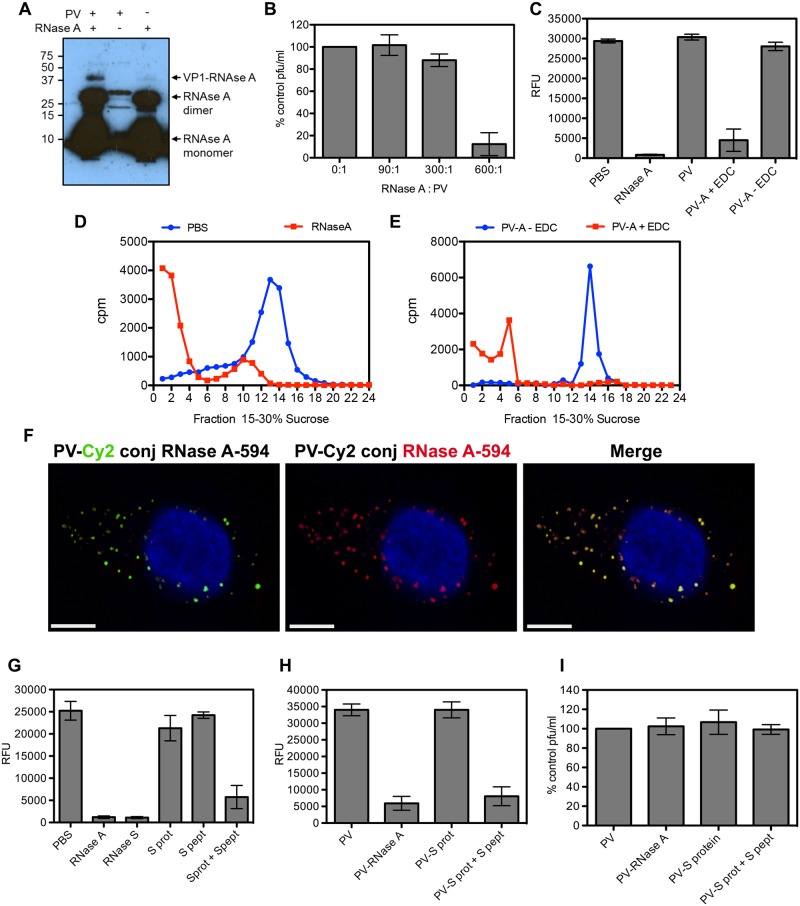
PV infectivity is not affected by covalent linkage of RNase A to the virus. **A)** RNase A is covalently linked to PV VP1. Conjugation reactions containing ^35^S-Met/Cys radiolabelled PV and/or RNase A (as indicated at the top of the image) were subjected to SDS-PAGE and western blot with antisera against RNase A. The major over-exposed bands correspond to RNase A monomer and dimer (indicated by arrows). Bands in the middle lane are the expected size for radiolabelled virus proteins VP1, VP2 and VP3. The upper band in the left hand lane is the expected size for RNase A covalently attached to VP1 (as indicated by arrow). Molecular weight standards (kDa) are shown on the left. **B)** Plaque assay of PV conjugated to RNase A (0, 90, 300, 600 molar ratio). Plaque forming units (pfu) were expressed as percentage of no RNase A control and data pooled from three independent experiments. **C)** Ribonuclease activity was measured by quantifying tRNA fluorescence (Relative Fluorescence Units, RFU) with Ribogreen in the presence of RNase A, purified PV, PV conjugated to RNase A with EDC (PV-A + EDC) and mock conjugation reaction (PV-A—EDC). **D)** Sucrose gradient profile of ^3^H-U-PV RNA (as in Fig 3E) uncoated *in vitro* at 50°C for 10 min in the presence of RNase A (1mg/ml) or PBS carrier control (representative of two independent experiments). **E)** Sucrose gradient profile (as in Fig 3E) of ^3^H-U-PV RNA from viral particles directly conjugated to RNase A with the cross-linker EDC (PV-A + EDC) or mock conjugated (no EDC, PV-A—EDC) uncoated *in vitro* at 50°C for 10 min (representative of two independent experiments). **F)** Representative image of HeLa Ohio cells infected with PV conjugated to Cy2 (green, left panel) and RNase A-DyLight594 (red, middle panel) fixed 15 min post infection. The degree of co-internalization (Merge, right panel) was measured for 10 random cells (R = 0.92 +/- 0.06 (SD). Nuclei were stained with Hoechst (blue). Scale bar is 5 μm. **G)** RNase activity (as in C) of individual and mixed components of the RNase S system. **H)** RNase activity (as in C and G) and **I)** virus titre (as in B) of PV conjugated to individual or mixed components of the RNase S system. (All data from three independent experiments with error bars showing standard error, unless stated).

The maintenance of both infectivity and RNA integrity in the presence of vast excesses of RNase strongly supports the hypothesis that the RNA of all endocytosed particles is protected. In order to confirm this hypothesis, we wished to specifically demonstrate the co-localization of internalized virions with RNase A by fluorescence microscopy. We therefore went on to examine the consequences of conjugating intact RNase A to PV particles. Covalent linkage of RNase A to virus proteins was performed using the zero-length cross-linker EDC and confirmed by western blot analysis of conjugation reactions containing RNase A alone, radioactively labelled (^35^S-Met/Cys) PV alone or both together using an anti-RNase A antibody (Fig 4A). Strong bands of monomeric and covalently linked dimeric forms of RNase A were evident, but in the presence of PV particles an extra anti-RNase A reactive band can be seen with an apparent molecular weight equivalent to RNase A linked to VP1. The infectious titre of PV was assayed following conjugation of RNase A at molar ratios of 90:1, 300:1 and 600:1, enzyme to virus (Fig 4B). Although the infectivity of particles conjugated using a 600-fold molar excess of RNase was severely compromised, 300-fold excess had a marginal effect on infectivity and 90-fold excess did not cause a measurable reduction in titre. The PV-RNase A conjugated particles produced using a 90:1 ratio were purified away from free RNase and shown to be highly catalytically active using a Ribogreen assay. In this assay, RNase mediated degradation of a tRNA substrate results in loss of intercalation-associated fluorescence (Fig 4C). In order to assess the susceptibility of PV RNA to RNase A during uncoating, ^3^H-U-PV was incubated at 50°C for 10 min to induce uncoating in the presence of 1 mg/ml RNase A or PBS carrier control. The entire reaction volume was then loaded onto a sucrose gradient to analyze the sedimentation profile of the ^3^H-U PV RNA. Fig 4D shows that a single peak corresponding to full-length PV RNA is present in the PBS sample. However, most of the radioactive signal from the RNase A-containing sample is found in the top third of the gradient, where RNA molecules smaller than the viral genome sediment. We repeated this assay with RNase A directly conjugated to PV using the EDC cross-linker or with a mock conjugation reaction (no EDC cross-linker) and the reactions were purified to remove unconjugated RNase A. We observed RNA degradation (Fig 4E; radioactive signal in the top third of the gradient) when uncoating is induced at 50°C for 10 min in the presence of conjugated RNase A, but not in the mock conjugation reaction. Finally, the co-localization of fluorescently tagged RNase A and PV following conjugation was assessed during the entry process by fluorescence microscopy (Fig 4F). RNase A was labelled with DyLight-594 and PV particles were labelled with Cy2 prior to conjugation. The PV-Cy2 conjugation was optimized to generate viral particles that were both visible by microscopy and also maintained 90% of the infectivity of the un-conjugated virus. Co-localization analysis of 10 random cells resulted in R = 0.92 +/- 0.06 (SD)(Fig 4F), strongly indicating that under conditions which did not reduce PV titre, all PV particles were covalently linked to RNase A. Again, we conclude from these results that viral RNA is transported into the host cell cytoplasm during the infection process by a mechanism that protects it from exposure to RNase in the endosomal lumen.

### PV infectivity is unaffected by covalent linkage of RNase S-protein to the particle before or after restoration of RNase activity by addition of S-peptide

Although high concentrations of RNase in the medium had no measurable effect on PV infectivity and particles which had been covalently-linked to active RNase A appeared to be fully infectious, these systems do not separate any effects on the virus due to conjugation alone from those that might be associated specifically with attachment of functional RNase molecules. To address this we made use of the products of subtilisin cleavage of RNase A. This protease cleaves the enzyme at a single residue to release a small peptide (S-peptide) from the remainder of the protein (S-protein), neither of which have RNase activity separately. However, when mixed, S-peptide and S-protein associate non-covalently to reconstitute enzyme function [40,41](Fig 4G). Similar restoration of RNase activity was demonstrated following the addition of S-peptide to S-protein that had been conjugated to virus particles and subsequently purified away from non-linked S-protein (Fig 4H). The advantage of using this two component system is that potential detrimental effects of conjugating protein to the virus particle can be separated from the additional consequences of infection in the presence of covalently linked functional enzyme. When coupling reactions were carried out with a range of S-protein to PV ratios there was minimal reduction of infectivity, 90% being retained, at S-protein to virus ratios of 100:1 and 500:1. Furthermore, there was no change in virus titre when S-peptide was added to restore RNase activity to S-protein conjugated virus particles (Fig 4I).

### The infectious process of the aphthovirus, ERAV, is resistant to RNase A

**Fig 5:**
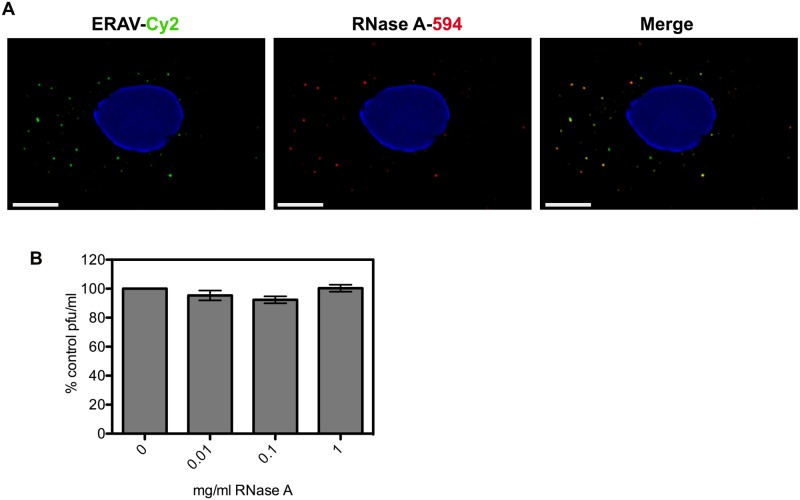
ERAV is co-internalized with RNase A but infectivity is not compromised. **A)** Representative images of HeLa Ohio cells infected with ERAV conjugated to Cy2 (green, left panel) and RNase A-DyLight594 (red, middle panel) fixed 15 min post infection. The degree of co-internalization (Merge, right panel) was measured for 10 random cells (R = 0.86 +/- 0.09 (SD). Nuclei were stained with Hoechst (blue). Scale bar is 5 μm. **B)** Plaque assay of ERAV in the presence of 0–1 mg/ml RNase A. Plaque forming units (pfu) were expressed as percentage of no RNase A control. Data are from three independent experiments with error bars showing standard error.

The uncoating and cell entry process for aphthoviruses is less well understood than for enteroviruses such as PV. Aphthovirus particles dissociate into pentamer subunits and release the RNA at pH values encountered during endocytosis [31,32]. This would be expected to expose the RNA to the contents of the endosome lumen. However, transiently formed empty particles, which have released their RNA, have been described for ERAV and may represent RNA delivery structures able to protect the RNA during the infection process [31]. To test this possibility we investigated the effect of high concentrations of RNase on the infectivity of ERAV. Similar to the results for PV, we found that the infectivity titre for ERAV was unaffected in the presence of concentrations of up to 1 mg/ml of RNase A (Fig 5B). To assess whether the infectivity observed in the presence of RNase could be explained by the segregation of virions and RNase A during infection, the co-localization of RNase A and virus particles within endosomes was probed by IF. The degree of co-internalization (Fig 5A) was measured for 10 random cells (R = 0.86 +/- 0.09 (SD) and provides compelling evidence for co-localization of virions with RNase A. The extent of the observed co-localization together with the nearly complete protection of viral infectivity demonstrate that, similarly to PV, RNA translocation in ERAV is insensitive to the presence of RNase A.

## Discussion

The mechanisms used by non-enveloped viruses to translocate their genomes across a cellular membrane to gain access to the cytoplasm are a matter of some dispute. Although there is some consensus that conformational changes in the virions that occur during internalization result in insertion of hydrophobic viral peptides into a membrane, there is disagreement about the roles played by these peptides in facilitating entry of the viral genome into the cytoplasm. In one model the viral genome is released from the virus into the lumen of an endosomal vesicle and is subsequently translocated across the endosomal membrane either passively, in a process similar to transfection, or by disruption of the endosomal membrane. In a second model the insertion of the viral peptides results in the disruption of the endosomal membrane and release of the viral particle into the cytoplasm where it subsequently releases its genome. In a third model the peptides form channels across the endosomal membrane, and the viral genome is released through these channels into the cytoplasm. The mechanism used could be expected to be specific for given families of viruses. Thus, the release of an intact particle (including the genome) through disruption of the endosomal membrane induced by viral peptides almost certainly occurs for the reoviruses, where the dsRNA genome itself is never released from the particle, and the cytoplasmic subviral particle serves as an RNA polymerase, releasing mRNA into the cytoplasm [42–44]. Penetration of subviral particles through disruption of the endosomal membrane mediated by viral proteins also occurs for adenoviruses [45] and polyoma and papilloma viruses [46]. For reviews of the field we refer the reader to [47–51].

Within the picornavirus family there are conflicting data concerning which of the entry mechanisms is used. For PV, and other members of the enterovirus genus, a number of lines of evidence suggest that the RNA is released through channels formed by the membrane-associated peptides. For PV, electrophysiology experiments have shown that the conformational changes associated with release of the VP4 and the externalization of the N-terminus of VP1 and their insertion into membranes results in the formation of channels and pores in the membrane [52], and genetic studies have shown that mutations in VP4 that prevent or alter the kinetics of RNA release also prevent the formation of channels or alter the properties of the channels [15]. Moreover, recent cryoelectron tomographic studies of putative translocation complexes produced using a receptor-decorated liposome model and conventional cryoEM images of virus complexes of virus with receptor-decorated large nanodiscs, provide strong support for the release of the viral genome through intact membranes without disrupting the membranes [22], and in the case of the nanodiscs model show density in the bilayer that could correspond to pores [33]. Biochemical experiments have demonstrated that recombinantly expressed VP4 from rhinovirus 16 is capable of inducing size specific pores in membranes [53,54], and cell-based studies have shown that rhinovirus 2 infection results in the induction of size selective pores in endosomes [55,56]. However, there is also evidence supporting the other two models. Thus, cell-based studies with rhinovirus 14 at very high MOI suggest that viral infection results in the disruption of endosomes [56], and the observation that acidification of aphthoviruses (such as FMDV and ERAV) results in the disruption of the virion to form pentamers and released viral genome *in vitro* and *in vivo* [31,32], suggest a model in which the RNA is released into the lumen of the endosome prior to translocation either by passive means or by particle induced disruption of the membrane.

In order to further probe the mechanism of RNA release of PV we have investigated the sensitivity of the RNA to RNase A both in the *in vitro* receptor-decorated liposome model and during infection. We show that RNA is translocated from the virus preferentially into the lumen of the receptor-decorated liposomes in a temperature-dependent fashion, that during this process the viral RNA is insensitive to the presence of excess RNase A, and that the release into the lumen of the liposome is highly directional with little or no RNA being released into the surrounding medium. We also show that the co-uptake of virus with RNase A into cells has no effect on viral infectivity or on the integrity of the bulk RNA, and that the infectivity remains insensitive to RNase when the virus is covalently coupled to RNase A at levels that guarantee that every virus particle has many copies of enzymatically active RNase. We believe that these findings rule out models in which any portion of the RNA is exposed to the lumen of the endosome. Moreover, we believe these experiments argue strongly against any model involving disruption of the endosomal membrane, because disruption of the endosomal membrane during infection would be expected to release RNase into the cytoplasm at levels that could locally overwhelm the cells ability to defend against cytoplasmic RNase and so result in fatal damage to the viral genome and death of the cell. Indeed, previous studies have shown that internalization of 100–200 nM of RNase A (*versus* the 75 μM used in our study) are sufficient to overcome endogenous cytoplasmic RNase inhibitors and kill cells [57].

Finally, both structural and fluorescence microscopy experiments demonstrate that PV encodes its own machinery to facilitate RNA translocation across intact membranes. Importantly, both our *in vitro* liposome based experiments and our cell based studies show that most/all of the viral RNA is protected from RNase digestion as it traverses the vesicle membrane. This strongly suggests that exit of the RNA molecule from the viral capsid is highly polarised, only occurring from a position adjacent to the membrane. Whether the polarisation of the RNA exit site implied by these findings is an innate property of the viral particle (i.e. that there is a unique site in the otherwise icosahedral virion that exists prior to membrane attachment and receptor-induced structural rearrangements) or is induced following association with the membrane is a subject for further investigations. These experiments, together with cryoEM structures and supporting genetic and biochemical studies, strongly favour a model in which the genomic RNA is transferred directly from the interior of the virus to the cytoplasm through virally encoded channels. The strong similarity of the biochemistry and structure of essential components of this machinery in disparate members of the enterovirus genus including enterovirus 71 [58], Coxsackievirus A16 [59], human rhinovirus 2 [60] and Coxsackievirus B3 [61] suggest this mechanism is conserved among all members of the enterovirus genus.

Although the observation that acidification results in the disruption of aphthoviruses into pentamers has led to the suggestion that the viral RNA is released directly into the lumen of an endosome, recent observations of the transient appearance of intact empty particles during the uncoating of ERAV [31] and FMDV [62] have led to the suggestion that full disruption into pentamers may be a late event that takes place subsequent to RNA release and translocation. We therefore decided to explore the RNase sensitivity of ERAV and showed that infection by this virus is also insensitive to co-internalized RNase, ruling out models in which the RNA is released into the lumen of the vesicle, and supporting the model in which the ERAV genome is translocated across intact endosomal membranes.

The observation that RNA release is insensitive to RNase in members of two distantly related genera of picornavirus (the enterovirus genus (PV) and the aphthovirus genus (ERAV)) that were once thought to release their genomes by very different processes, suggests that RNase resistant RNA translocation across intact membranes may be a general property of the entire family. Recent structural studies on Saffold virus 3 (SAFV-3) have suggested that the similarity may be extended to a third genus within the family, the cardioviruses. Thus, these studies have identified an altered (A) particle with an expanded internal volume, disrupted RNA-capsid contacts and pores in the capsid. *In vitro*, heat treatment of this A particle triggered genome release with the resulting empty capsid dissociating into pentamers [63]. Although further work is required to assess the interactions of the viral (or A) particle with membranes, the presence of an empty particle, albeit unstable, might represent an uncoating intermediate *in vivo*. Although such a general model for the release and translocation of RNA across membranes is attractive and supported by many experiments, more studies are clearly required to confirm that this general uncoating strategy is universal for all the picornaviruses since the rhinovirus HRV14 has been reported to disrupt endosomes during entry [56] at very high MOI. Although it is possible that HRV14 RNA translocation occurs with a mechanism different from that of HRV2, ERAV and PV, re-assessment of HRV14 uncoating in cells at lower MOI and in the presence of RNase A, as established here, would help shed light on HRV14 RNA translocation and ultimately its similarities or differences to the rest of the picornavirus family.

## Materials and methods

### Virus propagation and purification

PV type 1 Mahoney strain was grown in suspension HeLa S3 cells (maintained in the authors’ laboratory, Department of Biological Chemistry and Molecular Pharmacology, Harvard Medical School, and Faculty of Biological Sciences at the University of Leeds) and harvested by centrifugation. Cells were freeze-thaw lysed, and released virus was purified by differential centrifugation and CsCl density gradient fractionation [64]. To generate ^3^H-uridine labelled PV, HeLa S3 cells (4-5x10^8^) were infected with high MOI and 3 h post infection ^3^H-uridine (1 mCi; GE Healthcare) was added to the culture. To radio-label the virus proteins HeLa S3 cells were infected with PV in Met/Cys-deficient MEM (Sigma) and supplemented with ^35^SMet/Cys (10 μCi/ml; Perkin Elmer) at 2.5 h post-infection. The radio-labelled viruses were then purified as described above. ERAV was grown in adherent HeLa Ohio cells (Medical Research Council, Common Cold Unit, Salisbury, UK) and purified by sucrose gradient fractionation [31]

### Infectivity assays

The titres of PV and ERAV conjugated or not to fluorophores or RNase A were determined using a standard plaque assay. Serial dilutions of virus or virus complexes were added to HeLa Ohio cell monolayers and incubated for 60 min at 37°C on a rocking platform. The inoculum was replaced with growth medium containing 0.6% agarose (Sigma) and plates were incubated for 48 h. After fixing, plaques were counted and the titre of PV-RNase A was expressed as percentage of the control (unconjugated PV). To assess the effect of co-internalised RNase A on infectivity, 20–30 pfu were added to a monolayer of confluent cells in the presence of 0, 0.01, 0.1 and 1 mg/ml RNase A and incubated for 60 min at 37°C on a rocking platform. The RNase A-containing inoculum was then replaced with growth medium containing 0.6% agarose and incubated as described above.

### Liposome preparation

Phosphatidylethanolamine, phosphatidylcholine, sphingomyelin, cholesterol, and phosphatidic acid in chloroform (Avanti Polar Lipids) were mixed in molar ratios of 1:1:1:1.5:0.3, respectively [14,65]. Nickel salt of 1,2-dioleoyl-*sn*-glycero-3-{[*N*(5-amino-1-carboxypentyl) iminodiacetic acid] succinyl} (Avanti Polar Lipids), at a final concentration of 10% (wt/wt) was included in the lipid mix to allow for binding of the His-tagged receptor to the liposomes [16,19]. An Argon gas stream was used to evaporate the chloroform and produce a lipid film that was then dried under vacuum for 8 or more hours. Dried lipid film was rehydrated in 50 mM HEPES (pH 7.3) and 50 mM NaCl, YoPro-1 (Life Technology) at a ratio of 1:5000 (vol/vol), 1% glucose, at a final lipid concentration of 4 mg/ml. Liposomes were made by extruding rehydrated lipids through a membrane filter with 2.0 μm diameter pore size (Avanti Polar Lipids).

### Virus~receptor~liposome complex formation

The ectodomain of soluble PVRr with a six-histidine tag at the C terminus (sPVRHis), but without the cytoplasmic and transmembrane domains, was obtained as a gift from V. R. Racaniello (Columbia University College of Physicians and Surgeons, New York, N.Y.). 0.67mg/ml aliquots of sPVRHis were added to the liposomes at a 1:10 ratio (vol/vol). Receptor-decorated liposomes were diluted to 2 mg/ml in the same rehydration buffer (described above) with RNase A (Sigma) added (except when noted) to a final concentration of 50 μg/ml. 4 μl of 0.5 mg/ml virus were added to 26 μl of liposomes, and 30 μl samples were imaged.

### Covalent attachment of ribonuclease and fluorophores to virus

Conjugation reactions (20 μl) were set up with PV (1 μg,) and RNase A (0, 7.5, 25 and 50 μg/ml, corresponding to approx. 0, 90, 300 and 600 molar excess of RNase A) with EDC (1-ethyl-3-(3-dimethylaminopropyl) carbodiimide hydrochloride, 1 μg) and incubated at RT for 60 min. The reaction volume was then increased to 2 ml and concentrated to 200 μl in a Vivaspin column (50 kDa MWCO, Sartorius). PBS was added and concentration was performed again. In order to remove unconjugated RNase A, this procedure was performed 3 times. As controls, mock conjugation reactions were performed in the absence of EDC and RNase was removed by washing as above. The same conjugation conditions were used with virus and RNase S (Sigma). Fluorophore conjugation reactions were performed following manufacture’s protocol. PV and ERAV were labelled with Cy2 (GE Healthcare) and RNase A with DyLight-594 (Thermo Scientific). Free fluorophore was removed by centrifugation through a Vivaspin column as above and virus resuspended in PBS or HeLa Ohio growth medium.

### Fluorescence microscopy

For liposome-based assays wide-field images were captured using Olympus IX-71 inverted wide field microscopy with a Prior Lumen 200 Mercury Metal Halide lamp, Semrock BrightLine, and QImaging Retiga 4000R Monochrome Camera with RGB-HM-5 Color Filter and either a 20X, 0.4 NA objective (Fig 2A) or a 100X 1.35 NA oil-immersion objective (Fig 2C and 2D). For liposome-based assays with temperature ramping during imaging, one-color time lapses of YoPro encapsulated liposomes, settled on the bottom surface of Ibidi μ-slide channels (Ibidi, Munich, Germany), were imaged using a custom built Total Internal Reflectance Fluorescence Microscopy (TIR-FM) setup, attached to an Olympus IX-71 microscope (Olympus, Center Valley, PA) with an Olympus 100x, 1.45 NA oil-immersion objective. An Argon ion laser (Coherent, Santa Clara, CA) 488 nm line was adjusted to achieve HiLo TIR-FM [66]. Emitted fluorescence from the YoPro was collected with a 525/40 nm bandpass filter (Chroma, Rockingham, VT) and acquired at 0.25 second exposure with an Andor 885 electron-multiplying charge coupled device camera. Liposomes were imaged as the temperature was gradually increased from room temperature to 42°C. At the end of the temperature ramp, a separate field was imaged to evaluate the extent of photobleaching. For some control experiments, the images were acquired at 37°C. To reduce sample photobleaching due to production of free radicals, 1% glucose, 0.5 mg/ml glucose oxidase (Sigma-Aldrich), and 34 μg/ml catalase (Sigma) was added to the imaging buffer.

For cell-based fluorescence microscopy, HeLa Ohio cells (1.5 x 10^4^) were seeded on glass coverslips and incubated overnight. On the following day cells were cooled at 4°C for 30 min and the media removed prior to addition of the inoculum. The inoculum consisted of virus conjugated to the fluorophore (PV-Cy2; ERAV-Cy2) or PV-Cy2 conjugated to RNase A-DyLight-594. The viral inoculum was supplemented with Dextrans-10kDa-Alexa594 (Molecular Probes, 1 mg/ml) or RNase A-DyLight-594 (0.1 mg/ml). The supplemented inoculum was incubated on the cells for 30 min at 4°C, and then replaced by growth media supplemented with labelled dextrans or RNase A. Virus was allowed to internalise for 15 min at 37°C, then cells were washed and fixed in 4% paraformaldehyde and processed for IF. Images were acquired with a DeltaVision Deconvoluting microscope (Applied Precision) using a 60x objective and processed with SoftWoRx (Applied Precision) and Photoshop (Adobe).

### Image data analysis in the liposome assay

For the quantification of RNA-YoPro-1 signal in the wide-field images (Fig 2A), the images were masked to exclude regions at the periphery with no signal (a consequence of the low-magnification used in the experiment), the backgrounds were subtracted using a rolling-ball filter and histograms were constructed plotting the frequency of occurrence (y-axis) of pixels within the masked region with a given level of relative fluorescence. The resulting histograms were normalized to account for the different areas within the mask for the images obtained in the absence and presence of RNase and the normalized histograms were plotted together (Fig 2B). For fluorescent intensity measurements of frames (Fig 2E) from the movie used to construct the quantitative time course (Fig 2F), we selected a drift-corrected region of interest 19 μm^2^ from each image that exhibited uniform illumination. From the movie, we selected a continuous series of in-focus frames (ranging from five to 91 frames) at one min intervals. An image average was generated for each of the sets of in-focus frames, and the total intensity for the region of interest was calculated with ImageJ. The total intensity of each time point minus the total intensity at one min was plotted as a function of time post-initiation of temperature ramp-up (Fig 2F).

### RNA extraction and scintillation counting

Detached HeLa Ohio cells (10^6^) were cooled for 30 min at 4°C and ^3^H-U-PV (MOI = 1) was allowed to attach for 20 min at 4°C in the presence or not of RNase A (1 mg/ml). Samples were then transferred to 37°C and incubated for 30 min. Cells were pelleted and washed in cold PBS. The supernatant and washes were kept and analyzed by scintillation counting. The cell pellet was resuspended in Trizol (Ambion) and incubated on ice for 5 min. The RNA was extracted and cleaned (RNA miniprep kit, Zymo Research), then layered on top of a 5.5 ml 15–30% sucrose gradient (0.1% SDS, 0.1 M sodium acetate, PBS) and spun at 50k rpm for 1 hour 40 min at 20°C in a SW55Ti (Beckman). Gradient fractions (~250 μl) were collected with a microdispenser syringe from the bottom of the tube using a cannula and flexible tubing. Scintillation counting fluid (3 ml) was added, the tubes vortexed and cpm counted (GE Healthcare).

### Ribonuclease activity

Ribonuclease activity was measured by quantifying tRNA fluorescence with Ribogreen reagent (Life Technology). Virus, RNase (A, S, S peptide, S protein) and virus-RNase conjugates were combined in PBS with 0.2–1 μg tRNA and Ribogreen (1 in 200 dilution) and fluorescence (485/520 nm) was measured using a BMG Optima plate reader. For RNase A activity during *in vitro* uncoating, ^3^H-U-PV was incubated at 50°C for 10 min in the presence or not of RNase A (1 mg/ml). Immediately after incubation, the entire reaction volume was loaded onto a 15–30% sucrose gradient and processed as above. Similarly, ^3^H-U-PV conjugated to RNase-A was uncoated *in vitro* and the RNA analyzed on a sucrose gradient.

## Supporting information

S1 Movie Real time RNA translocation into receptor decorate liposomes. Movie showing RNase resistant translocation of viral RNA from virions into YoPro-1 containing liposomes as complexes of PV with receptor-decorated liposomes are gradually warmed from room temperature to 42°C. The red channel shows labeled beads, using focusing aids. The green channel shows the YoPro-1 signal as it interacts with the viral RNA entering the liposome. The last several noticeably brighter frames of the movie show a separate field of view where the label has not undergone photobleaching. The data shown in this movie were used to produce Fig 2E and 2F in the parent manuscript.
(M4V)
